# Cost of hospital care for HIV/AIDS infected patients in three general reference hospitals in Lubumbashi, DR Congo: prospective cohort study

**DOI:** 10.11604/pamj.2013.15.76.2638

**Published:** 2013-06-26

**Authors:** Henri Mundongo Tshamba, Clarence Mukeng a Kaut, Nono Mulubwa Kyalubile, Alphonse Kaij Kakambal, Grevisse Ditend Yav, Françoise Malonga Kaj, Didier Vancaillie

**Affiliations:** 1 University of Lubumbashi, Lubumbashi, DR Congo; 2 Nursing care, ISTM-Lubumbashi, Lubumbashi, DR Congo; 3 School of management's University of Liege, center for Research on Corporate Performance, Liege, Belgium

**Keywords:** Healthcare costs, HIV AIDS, Predictive factors for healthcare consumption

## Abstract

**Introduction:**

This article analyses the composition of healthcare costs for HIV/AIDS infected patients in a country with limited resources and attempts to identify the factors that influence these costs. The aims are to calculate medical care costs, analysing how they vary depending on patients’ income, and to evaluate the factors explaining healthcare consumption.

**Methods:**

This is a prospective cohort study focusing on patients who were admitted to hospital for a short stay between January 2010 and June 2011, before their integration into a specialised program. The patients were selected randomly. Free consent was obtained from all participants. Data were analysed using the SPSS 19.0 software. The significance threshold was set at 5% and the CI (Confidence Interval) at 95%. We used Kruskal-Wallis tests, Fisher's exact test and multiple linear regression.

**Results:**

We monitored 209 patients. Their average age was 36.37 years (SD: 8.72). The sex ratio was 0.58 and the women patients were generally younger than the male ones (p=0.011). The overall cost of healthcare amounted to $US 41,922. The cost of Antiretroviral Therapy represented 21.6% ($US 9,045). The price of para-clinical examinations represented 46% ($US 19,136) of the overall cost. The patient's average monthly income was $US 157.40 whereas the average direct cost per patient was$US 201.45. Both monthly income (t=4.385; p=0.0000) and education level (t=3.703 p=0.0003) were statistically significant predictive factors for healthcare consumption. The medical care costs for patients with opportunistic infections were nine times higher than those for patients who presented none. The presence of opportunistic infections increased healthcare consumption by approximately 31$ US (CI 95%: 15-46.9).

**Conclusion:**

The average direct cost for patients on each short-term stay was higher than the average monthly income. To be able to access the necessary services, the patients need additional resources, which are derived from various sources. Monthly income and the level of education were both statistically significant predictors for healthcare consumption. The analysis allows us to extend the study by using different analytical accounting approaches such as by case and by pathology.

## Introduction

HIV/AIDS is a major health issue and in some countries the number of patients is increasing exponentially. We found many types of influences: cultural, economic and political [1, 2]. Twenty-five million people live with HIV in sub-Saharan Africa and, in the DRC, the HIV/AIDS epidemic has reached a generalised level; its prevalence fluctuates between 1% and 15% [1–6]. AIDS is both an urgent problem and a developmental issue with longer term implications. Despite the increase in funding and in political engagement and despite the progress made to improve access to treatment, the epidemic is progressing faster than the world is fighting back [3, 4, 7].

A powerful combination of at least three drugs, termed Highly Active Antiretroviral Therapy (HAART) exists and is widely used [6–13]. The use of tri-therapy has led to a striking decrease in morbidity and mortality in patients infected with HIV [13, 14]. Nevertheless, this progress has been made at the cost of important expenditures, estimated between 10,000 and 15,000 $US per patient per year in the US [8, 10, 15]. Several studies have estimated the monthly healthcare costs to patients and have studied the relationship between these costs and patient CD4 counts [7]. However, the demographic and socio-economic impact of AIDS is important especially in terms of the mortality affecting labour force, which is much needed for country's development, and in terms of the overall healthcare expenses, which remain precarious [2, 4, 16].

A macro-economic analysis of the DRC's national and international spending on the fight against AIDS showed that 96,365,322 $US was spent (38% of the amount needed); 11% of the funding was provided by households [2]. Considering that 71% of the population live under the poverty threshold, the national per capita income is 300 $US, and the country's human development index is 0.389 according the UNDP, a precise measure of patient expenditures is due [2, 17].

Indeed, an understanding of the costs for hospital care associated with AIDS will not only allow the economic consequences of HIV infection for household or state budgets to be measured but will also provide the decision makers with precise information that they can then use to allocate financial resources efficiently. Our approach fits precisely into this framework.

## Methods

This observational study was for analytical purposes. The study used an activity-based costing (ABC) method to calculate the cost of the patient's course [18–22]. The English authors have shown that the chosen hospitals are situated in favourable environments and are good candidates for applying the ABC method used to calculate costs [23]. The ABC method is being used increasingly in hospital settings as an alternative to the traditional costing method [24–28].

This prospective cohort study focused on patients who were admitted to hospital for the first time and who agreed to participate. The charges paid for by the patients were recorded in accounting documents. They included the costs of hospital stays, drugs (ARV and No-ARV), Para-clinical examinations, and the costs associated with medical or nursing procedures.

During the selection process, an informed consent was obtained, after which 209 HIV seropositive patients were randomly selected to be monitored in three general reference hospitals in Lubumbashi (Kenya general hospital of reference, Sendwe provincial hospital and academics clinics of Lubumbashi).

Data were collected in a double-blind manner using Epi Info 3.5.3 2011 software. We also used SPSS 19.0 for data analysis. The significance threshold was set at 5% and the CI at 95%. Kruskal-Wallis tests, Fisher′s exact test and progressive linear regression were used to test the relationship between the variables found in this study.

## Results

Between January 2010 and June 2011, we interviewed and monitored 209 HIV-infected patients who were admitted to hospital for the first time. Our study focused on healthcare costs that did not get into a specialised program or were not subsidised by Non-Governmental Organizations (NGOs) or the Ministry of Health. The socio-demographic characteristics of the patients are shown in Table 1. For all patients, the average stay was seven days. The cohort was dominated by female patients; the age difference between the two sex groups was significant (p=0.011). The breakdown of the resources consumed by the patients during their hospital stay enabled us to describe the composition of the various costs and identify the hospital services paid for by this homogeneous group of patients. These are presented in Table 2.

**Table 1:** Baseline characteristics of the 209 HIV/AIDS Infected patients

	Values Patients (n=209)	S.D	P Value
Average Age (All sex: Minimum-Maximum)	36.37 (17-58)	8.72	
Sex ratio	76/133 (0.57)		0.011
Married	87 (41.6%)		0.040
Sgle	63 (30.1%)		
Divorced	42 (20.1%)		
Widowed	17 (8.1%)		
Average income declared	157.40 (CI 95% :144.15-170.65)	94.26	
Average health care cost per patient (CI 95%)	201.45 (CI 95% :196.62-206.28)	34.38	
Average length of hospital stay	7 (1-20)	2.7	
**Health Insurance and lifestyle**			
Patients no insured	209 (100%)		
Patients insured	0 (0%)		
Hétérosexuels	190 (91%)		
**Patients classification according to CD4+ Strata and Treatment type**			
Median CD4+ / µL (Range)	200 (10-650) IQ =106		
< 200 Cells / µL	96 (45.9%)	0.510	
200-299 Cells / µL	87 (41.6%)		
≥ 300 Cells / µL	26 (12.4%)		
Patients receiving HAART	201 (96.6%)	0.006	
Median CD4+ cells count for patients receiving HAART	200 (10-650)		
Median CD4+ cells count for patients without HAART	376 (350-412)		
Presence of opportunistic infections	1.9%)		

**Table 2:** Healthcare costs in $ US by hospital service for 209 patients and CD4+ count

CD4+ Strata	Nb	Total Cost	HAART Costs	No HAART Costs	Physician/ Nursing Costs	Hospital Costs	Lab/Radio Exam Costs
< 200 Cells/µL	96	$18750	$4320	$2376	$2256	$1286	$8512
200-299 Cells/µL	87	$18233	$3915	$2649	$2219	$1280	$8170
≥ 300 Cells/µL	26	$4939	$810(18)	$701	$648	$326	$2454
**Total**	209	$41,922(10)	$9,045(21.6)	$5,726(13.7)	$5,123(12.2)	$2,892(6.9)	$19,136(46)

The overall cost of the patient's medical care reached ($US 41,922. The price of Para-clinical examinations represented 46% ($19,136) of the total cost. These results show that expenses were eight times greater for patients whose CD4 count was below 300 cells /µL than for patients who had a CD4 count over 300 cells /µL. The results indicate that the overall cost was greater than the cost to the institutions. This observation relates to the fact that these institutions limit their spending to only a portion of the services that the patients need. The remainder of the prescribed services are funded directly by the patients (drugs, other healthcare equipment...) outside the hospital. This leads to patient dissatisfaction and further increases the costs of health care or hospital care fees. The composition of healthcare costs enabled the hospital services that were financed by the patients to be identified. A breakdown of these costs provided information about the relationship between disease stage and care costs and allowed those involved to become aware of this relationship as well as of the economic effects of HIV infection on patient income. Given these conditions, are patients able to afford healthcare and benefit from medical support using only their monthly income? The results indicate that 124 patients (62.9%) had an average monthly income below 200$US. These results demonstrate beyond doubt that this group could not afford the average cost of medical care during a short-term hospital stay, which amounted to 201.45$US.

The results summarised in Table 3 on the specification model for linear regression as used to analyse variables that could explain healthcare consumption. Monthly income (test t=4.385; p=0.000) and patient education level (test t=3.707; p=0.000) were statistically significant predictive factors for healthcare consumption. Considering the observation that disease stage and the presence of opportunistic infections are linked (x^2^=14, 3118; p=0.0008) we built the following in Table 4 in order to measure the impact of opportunistic infections on healthcare costs. From these results we can see that medical care costs for patients who carried opportunistic infections were nine times higher than for patients who presented no opportunistic infections.

**Table 3:** Model coefficients

Model	Non-Standardised coefficients		Standardised coefficients	t	P
	β	Std. Error	β		
(Constant)	182.349	4.5161		40.378	0.000
Patient's monthly income	0.121	0.025	0.333	4.926	0.000
(Constant)	159.948	7.466		21.422	0.000
Patient's monthly income	0.106	0.024	0.291	4.385	0.000
Education level	11.910	3.217	0.246	3.703	0.000

**Table 4:** Breakdown of medical care cost and presence of opportunistic infections

Opportunistic Infection	Patients (N)	No ART Costs	Physician/Nursing Costs	Hospital Costs	Lab/Radio Exam Costs
Presence	192	5378 (28)	4708 (24.5)	2682 (13.9)	17841(92.9)
Absence	17	348 (20)	415 (24.4)	210(12.3)	1295(76.17)
Total	209	5726	5123	2892	19136

## Discussion

The overall medical care costs for these patients amounted to $US 41922. The cost of Para-clinical examinations represented 46% ($US 19,136) of the overall cost. The cost of antiretroviral drugs represented 13.7% ($US 5,726), non-antiretroviral drugs 21.6% ($US 9,045), healthcare procedures 12.2% ($US 5,123) and hospital stays 6.9% ($US 2,892). The patients’ average monthly income was $US 158.40 (CI 95%:144.15-170.65) while the average direct cost per patient was $US 201.45. In Spanish, the total cost for HIV related health care assistance was Euro 739,048. The costs related to admission was Euro 150,766.60, 8,631 Euro per first visit and Euro 49,199.40 per successive visit and 5,085.10 per day care visit; Euro 14,920 per outpatient surgery; Euro 7,655.70 per ER visit, Euro 491,342.40 per antiretroviral treatment Ray et al. (2006) observed that $US 10,500 were spent on highly active antiretroviral (HAART) drugs per patient per year. The cost of medical and clinical procedures represented $US 359 per patient per year or 2% of the overall expenses. The cost of drugs was thought to represent 71-84% of yearly expenses [7].

Our results indicate that the total cost of medical care was eight times higher for patients who had a CD4 count below 300 cells/ µL than for those who had a CD4 count over 300 cells/ µL. The differences were predominantly due to the costs of Para-clinical examinations, no-antiretroviral drugs, nursing or medical procedures and the hospital stay. The cost of drugs (both Antiretroviral Therapy and No-Antiretroviral Therapy) represented 35.2% of the overall cost.

Our observations lead to the hypothesis that patient adherence to tri-therapy will improve immune recovery and CD4 count and will lead to a decrease in the cost of healthcare. This is in agreement with observations made by Ray et al. (2006), who noticed that a decrease in the CD4 count was associated with an increase in spending; this increase was statistically significant for patients whose CD4 counts fell within 50-199 cells/ µL (p=0.003).These observations lead us to confirm the economic advantage of tri-therapy, which is currently recommended as treatment for HIV/AIDS patients [6, 7, 29–32].

Worldwide spending on AIDS has increased from 5 billion $US in 2003 to 20 billion $US in 2007 with most of the expense directed towards prevention campaigns and patient care in countries with low and middle incomes [32]. In the US, yearly expenditures per patient ranged from 10,000 to 15,000 $US [7, 15]. Because a substantial proportion of the medical care costs must be paid by the patient, we wanted to identify which factors influenced healthcare consumption: our results indicated that monthly income (Test t=4.385 p=0.0000) and education level (Test t=3.703, p=0.0003) are statistically significant predictive factors. These observations suggest that, if the patient's monthly income increased by 1$US, there would be an impact on healthcare consumption of about 10 cents (CI95%: 0.052 - 0.145). Education level had a positive effect on healthcare consumption, increasing it by about 13.5 $US (CI95%; 7 - 19.7). The presence of opportunistic infections increased healthcare consumption by approximately 31 $US (CI95%; 15 - 46.9)). In Sudan, the total cost associated with management of tuberculosis was significantly higher for HIV positive as compared with HIV negative TB patients ($US 105.08 to 73.92 p=0.003) [33]. In Mexico, the estimated model explains about 45% of the variation in costs. Additional education is significantly and positively associated with cost. Increasing age is also associated with higher costs [34]. But Pedram S et al. had observed that given a patient's clinical health status, a higher educational level and a stable partnership were associated with greater ability to work [35–37].

Socioeconomic characteristic may influence the cost effectiveness of health care interventions in HIV-infected patients. Information about a HIV patients’ serostatus provides small expected benefits to health care workers ($US 3.34), but HIV seropositive test result provides large expected benefits to the patient ($US 11,202) and to the patient's sex partners ($US 5,271) [38]. In addition to informing us about the costs themselves, decomposing the expenses into several components allowed us to identify which hospital services are financed by the patients. A breakdown of the costs provided important information about the cost differences for patients at different stages of the disease. This relationship, along with the economic consequence of HIV infection on the patient's income, can be made clear to decision makers.

An examination of the patient's immune status revealed that: the median CD4 count for the whole cohort was 200/µL (10-650; IQ: 106); 45.9% of patients had CD4 counts below 200/ µL. There was a relationship between a patient's CD4 count and his/her disease stage (likelihood ratio=134.034, p=0.000). These results are similar to those obtained by other authors. Clumeck et al. observed a median CD4 count of 165/ µL and a viral load (VL) of 5.2 log cp / ml [1]. In a study carried out in South Africa, Wandeler et al. found that HIV patients had a median CD4 count of 172/ µL (95-267) [2].

Our results demonstrated that the average age of our cohort was 36.37 (SD 8.72). The sex ratio was 0.57 and the female population was younger than the male population (p=0.011). While studying a cohort of patients eligible for treatment in Lubumbashi, Clumeck et al. found that 72% of patients were female while 28% were male. Wandeler et al. reported that the median patient age was 38 in a study conducted on HIV seropositive patients in South Africa [1, 2]. In their study in Benin, Zannou et al. found that the average age of HIV/AIDS patients was 37 ± 2 years [39]. These data are very similar to our own results.

Altogether these observations indicate that, on the one hand, this disease is increasingly affecting women, and on the other hand patients are generally fairly young.

We noticed that patients tend to be diagnosed at an advanced disease stage (stages II or III for 87.5% of them). This fits with observations from authors who found that patients tend to seek medical help when immunodeficiency has become pronounced and when they are at a very advanced stage of infection [32– 33, 35]. This highlights the need to intensify screening campaigns, raise awareness in afflicted communities and promote early admission of HIV/AIDS patients. These measures will not only improve the clinical well-being of patients and decrease contagiousness but will also reduce healthcare costs.

## Conclusion

HIV/AIDS has become a complex problem with consequences that go beyond issues of public health to include all sectors of socio-economic development. For a short hospital stay, the average cost of medical care per patients is greater than the average monthly income. To be able to access the necessary services, the patients need additional resources, which are derived from various sources. Monthly income and education level are both statistically significant predictive factors for healthcare consumption; however, the presence of opportunistic infections increases the cost of medical care.

To fight stigma and discrimination that will affect female patients, it is important to take action against the numerous factors that contribute to their vulnerability regarding HIV/AIDS, such as gender inequalities, socioeconomic factors and sexual violence.

It is important to extend studies that have looked at cost/effectiveness of various healthcare programs for HIV infected patients. This analysis allows us to objectively consider the use of different analytical accounting approaches such as by case and by pathology.
